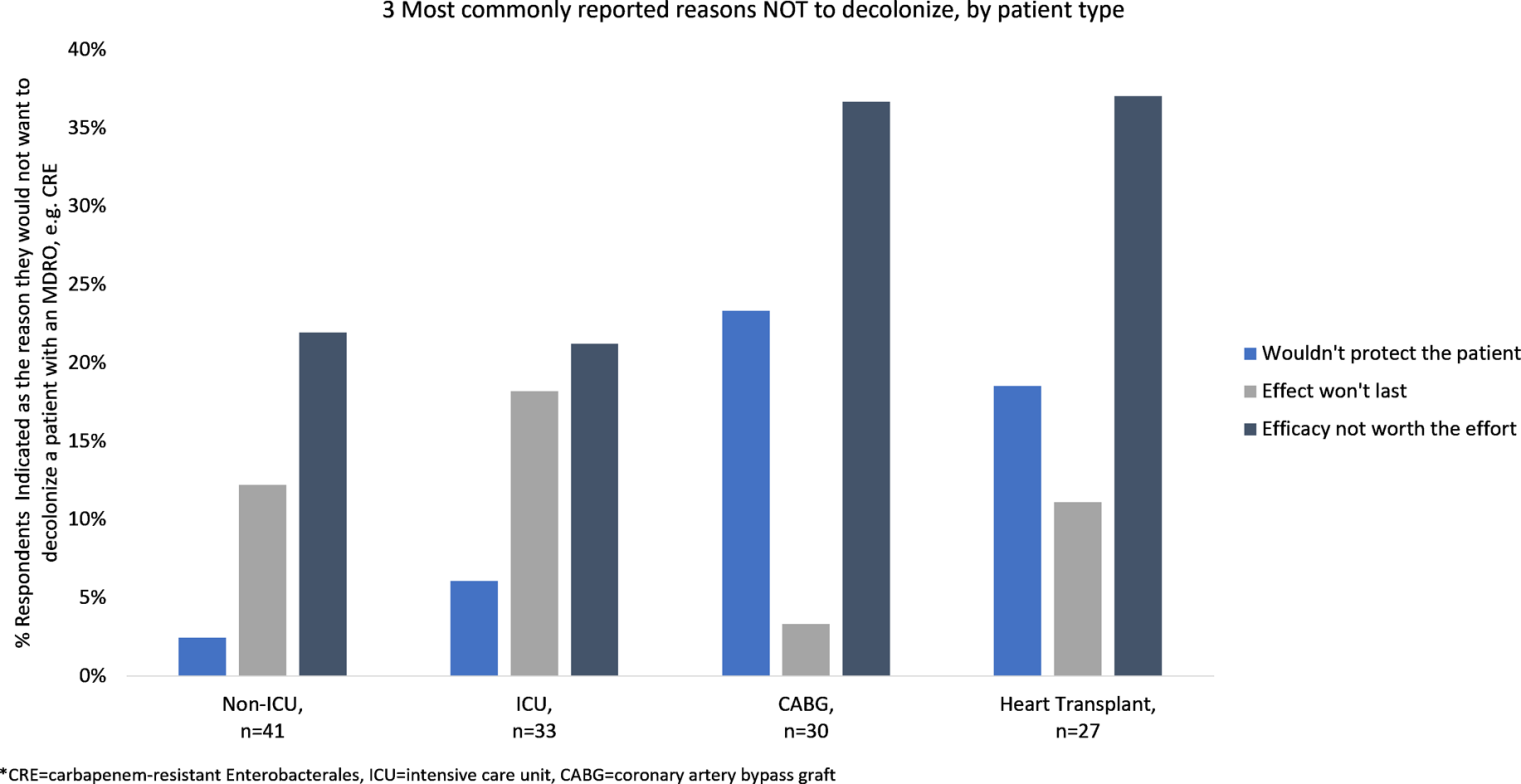# 174 Changes in Antibiotic Use in the Outpatient Setting – United States, 2019 and 2023

**DOI:** 10.1017/ash.2026.10574

**Published:** 2026-06-23

**Authors:** KC Coffey, Trudy Grossman, David Banach, Anthony Harris, David Hooper, Susan Huang, Erica Shenoy

**Affiliations:** 1 Massachusetts General Hospital; 2 CARB-X; 3 Unviersity of Connecticut School of Medicine; 4 University of Maryland School of Medicine; 5 University of California Irvine School of Medicine; 6 Massachusetts General Hospital and Mass General Brigham

## Abstract

**Background** Decolonization decreases risk of healthcare-associated and post-discharge infections. Most decolonization data are derived from methicillin-resistant Staphylococcus aureus (MRSA) studies, although results have been extended to other multidrug-resistant organisms (MDRO). Understanding MDRO target outcomes and preferred product characteristics may inform new decolonization protocols, products, and strategic integration into infection prevention practices. Methods We surveyed 134 Society for Healthcare Epidemiology of America (SHEA) Research Network-affiliated US healthcare facilities on MDRO surveillance, isolation, and deisolation practices. The survey asked about decolonization practices, needs, and gaps. The survey was administered via REDCap from 1/7/25-2/25/25, and responses were de-identified. Frequencies and proportions were analyzed in REDCap and Excel. This survey was considered nonhuman subjects research by the Mass General Brigham Institutional Review Board. Results Of 134 facilities surveyed, 52 (39%) completed the decolonization section of the survey. 38/52 (73%) facilities reported performing some form of decolonization. Of these, 87% said the most important decolonization outcome is reduced risk of progression to infection. Lab-based clearance (8%) and reduced risk of transmission (5%) were considered less important. Using carbapenem-resistant Enterobacterales (CRE) as an example of an MDRO without established decolonization products or protocols, respondents’ interest in decolonization increased with patient vulnerability to invasive disease (Figure 1). Preferred decolonization agent characteristics varied by patient population, e.g. a majority selected transmission interruption in the non-intensive care unit compared to patient-specific efficacy in the transplant population (Figure 2). Half of the surveyed facilities reported having unmet decolonization needs. Across all patient types, the most commonly reported reasons not to decolonize were “efficacy not worth the effort”, “wouldn’t protect the patient” and “effect won’t last” (Figure 3). Approximately one quarter of respondents said they would not want to decolonize for “other” reasons including, “no established protocol” and “unclear evidence”. Conclusions Facilities reported valuing decolonization as a means to prevent progression to infection, but transmission prevention was rarely selected as an important outcome of decolonization. Due to reported preferences of agent characteristics by patient population, there may be opportunities for development of population-specific products. Product design should prioritize decolonization agents that are easy to administer, highly effective, and provide long-lasting impact. Evidence-based, standardized decolonization protocols, particularly for gram negative MDRO, are needed to address barriers and support decolonization in prevention of transmission as well as infection.

**Figure:**
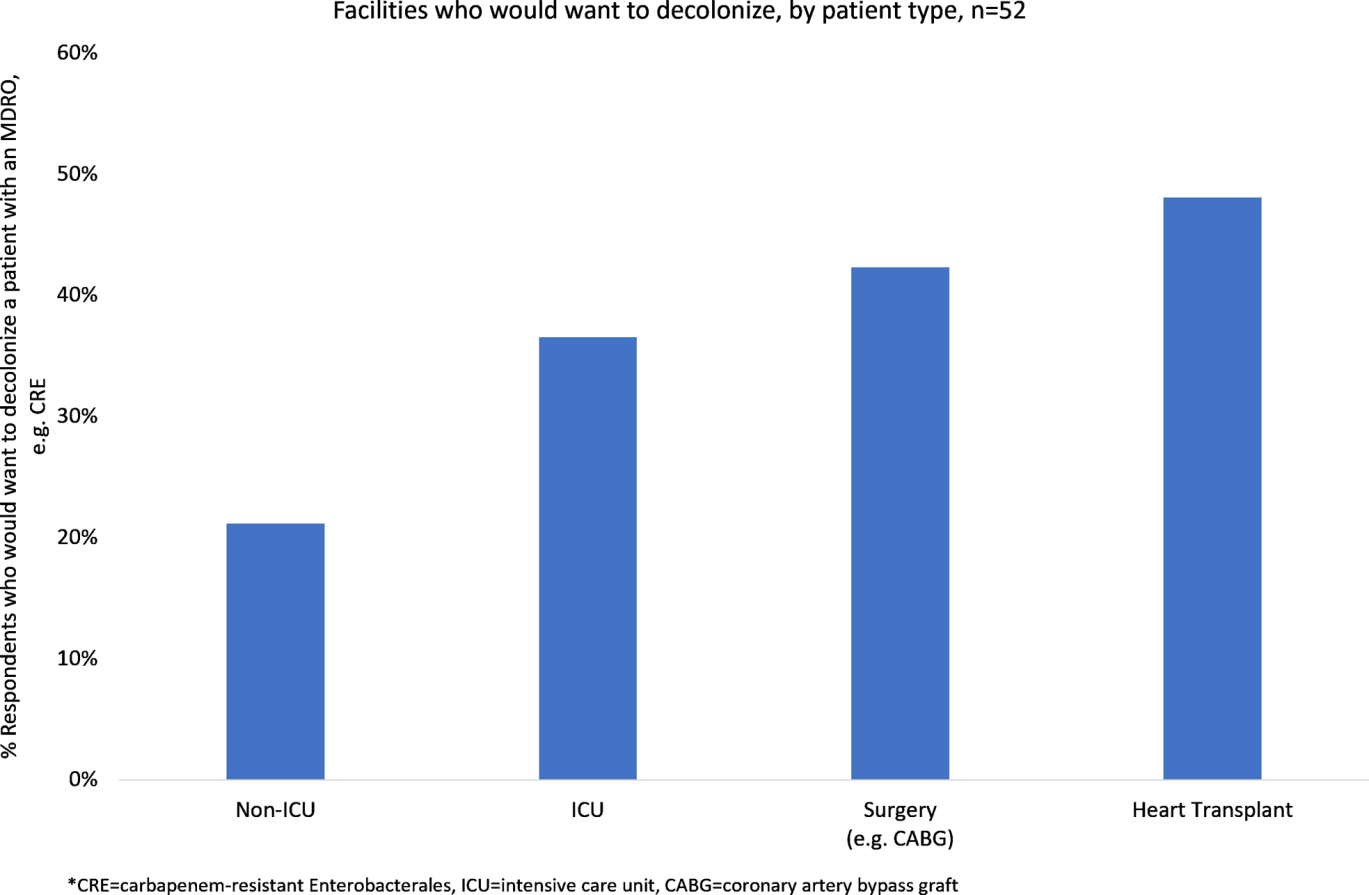

**Figure:**
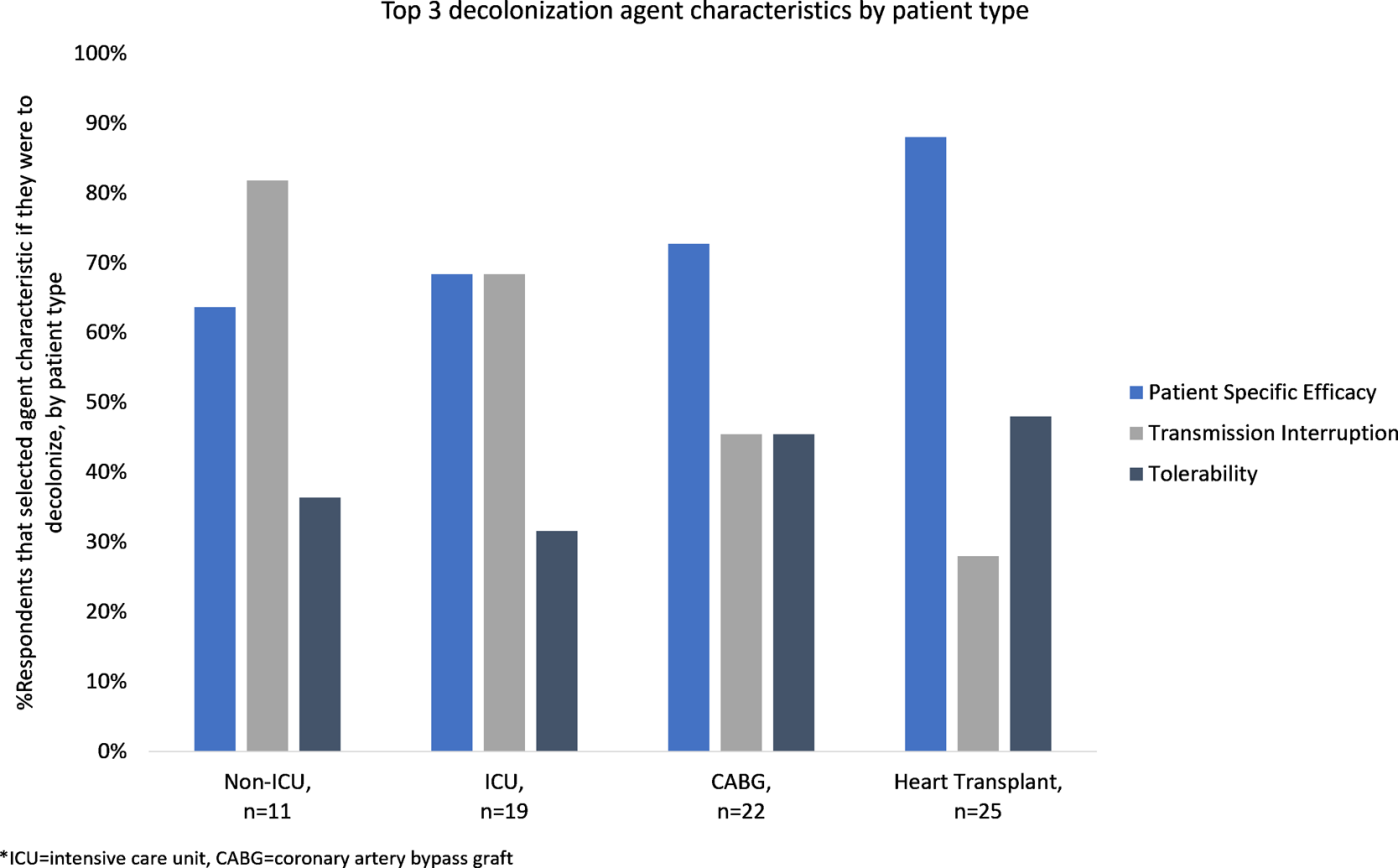

**Figure:**